# Wool or Cotton?

**Published:** 1911-11

**Authors:** 


### Wool or Cotton?

THE season is here when the physician is called upon for advice—or may find the way to offer it unasked—as to clothing. From ancient times, woolen has been the accepted fabric for winter undergarments and it is only recently that red has lost its favor as both literally and symbolically superior in warmth. However, woolen and cotton garments placed in a tub, behave in such a way as to suggest that heresy may go a step further and relegate the former to the past. If wool will not except with difficulty, absorb water from a tub, how can it absorb perspiration? And, while a warm, sweaty body gives the subjective impression of conservation of heat, it is to be seriously questioned whether exposure to external cold while the body is coated with perspiration, will not produce chill. On the other hand, cotton or linen underwear, of heavy weight, will absorb a considerable amount of water, allow it to evaporate gradually and thus keep the skin dry.

So much for theory. As to the practical side, we can only give, for what it is worth, four years of comfort in cotton garments and a half day of suffering from cold feet on account of a temporary return to woolen socks.

We are inclined to believe, however, that old persons, with relatively inactive skins and not liable to prolonged outdoor exposure after perspiring, might better wear woolen.

It is a curious fact that laborers frequently wear in summer as heavy undergarments as professional and business men wear in winter. We have never been able to appreciate their philosophy. Within the last generation, the woman who wore summer underwear the year around, seems to have given place to a more sensible type. But we can't help wondering what the girls who have been wearing winter .cloth coats this summer, will do in December. Will there be a rise in incidence of catarrhal troubles?

